# Healthy and Balanced Nutrition for Children through Physical Education Classes

**DOI:** 10.3390/life11070678

**Published:** 2021-07-11

**Authors:** Rubén Trigueros, Sergio González-Bernal, Jerónimo J. González-Bernal, Raquel de la Fuente-Anuncibay, José M. Aguilar-Parra

**Affiliations:** 1 Hum-878 Research Team, Health Research Centre, Department of Psychology, University of Almería, 04120 Almería, Spain; 2 Department of Psychology, University of Burgos, 09001 Burgos, Spain; sbernall@ubu.es (S.G.-B.); jejavier@ubu.es (J.J.G.-B.); raquelfa@ubu.es (R.d.l.F.-A.)

**Keywords:** Mediterranean diet, school, self-determination, physical education, theory of planed behaviour

## Abstract

*Introduction*. In recent years, the rate of childhood obesity has been on the rise, currently standing at levels close to 20%. This means that one in five children is more likely to suffer from cardiovascular or metabolic diseases. Physical Education classes are therefore an ideal way to raise awareness among children and their families about healthy and balanced eating habits. *Method*. A total of 113 primary school students, aged 9–12 years, participated in the study. In order to analyze the data, a structural equation model (SEM) was used to analyze the influence between the variables. *Results*. The SEM results revealed that a controlling social context showed a negative prediction of psychological need satisfaction and a positive prediction of frustration. However, an autonomy supportive social context showed a negative prediction of psychological need satisfaction and a positive prediction of psychological need satisfaction. Frustration of psychological needs was negatively related to motivation, whereas satisfaction was positively related to motivation. In turn, motivation was positively related to each of the factors of the theory of planned behaviour. Finally, intention to follow a healthy diet was positively related to the Mediterranean diet. *Discussion*. These results revealed the importance of social context and physical education classes in the adoption of a balanced diet.

## 1. Introduction

According to the latest report of the Spanish Society of Cardiology, nearly 38% of Spanish children aged 6–12 years are obese or overweight [1,2]. In addition, abdominal obesity affects three out of ten children, especially boys [1]. These data reflect a clear increase in childhood obesity rates in Spain in recent years, see [3,4]. In this sense, childhood overweight and obesity are associated with a higher probability of becoming obese adults and with a higher risk of suffering from diseases, such as type 2 diabetes, cardiovascular disease or certain types of cancer in adulthood [5,6]. For this reason, Physical Education (PE) classes can play a fundamental role in promoting healthy lifestyle habits, as their objectives include healthy and balanced nutrition and the adoption of active habits during leisure time [7]. In addition, the influence of parents and teachers on the dietary behaviour and physical exercise of young people is fundamental [8].

There are several theories that have analyzed human behaviour, among them, the Self-Determination Theory (SDT) [9,10]. This theory states that the social context (e.g., teachers, parents, etc.) exerts a very important influence on the individual’s behaviors, which can be through two very different styles: autonomy supportive and controlling [11,12]. Autonomy support refers to the social context promoting the individual’s mental and physical development through personal initiative [13,14]. In contrast, the controlling style promotes the physical and mental development of the individual but based on the use of coercive means and external pressures, which prevents personal initiative [13,14]. These interpersonal styles exert a very important influence on the satisfaction or frustration of basic psychological needs, which are basically internal psychological nourishments, present in all human beings, and which are necessary for the correct social and psychological development and well-being of people [15,16]. Thus, those people who perceive autonomy in their decision-making, competency in their actions and support and integration in their social reference group will feel a satisfaction of their psychological needs [16], which is related to commitment and permanence in the activity [17]. However, if people experience a feeling of abandonment, low success in their actions and a lack of decision-making, they will experience a frustration of their psychological needs [16,17], which is related to disengagement and disengagement [16,17]. Thus, those who experience frustration of their own psychological needs will show a greater predisposition towards controlled motivation and even demotivation [18]. On the other hand, those who have their psychological needs satisfied will show a greater predisposition towards autonomous motivation [19].

Studies focusing on self-determination theory at the primary school stage are certainly scarce. However, it is a stage that is fundamental in the adoption of habits during adolescence and adulthood [20]. Among the studies that have analyzed the basic psychological needs and motivation of students at the primary school stage, the one by Van-Aart, Hartman, Elferink-Gemser, Mombarg and Visscher [21], which showed positive predictability between satisfaction of basic psychological needs and intrinsic motivation, stands out in relation to Physical Education classes. Similarly, a study by Patón, Fernández and Nemiña [22] showed the positive correlation between basic psychological needs satisfaction and motivation and these, in turn, with students’ enjoyment and involvement during PE lessons. However, these studies focusing on the primary school stage have not analyzed the effect of the social context (parents and teachers) on basic psychological needs, despite the influence they exert on children’s behavioral patterns. Similarly, these studies have not taken into consideration the effect of frustration of students’ basic psychological needs on motivation nor have they taken into account students’ behavioral patterns in relation to the basic objectives of the area of PE.

The Theory of Planned Behaviour (TPB) is one of the main theoretical models that tries to explain the predictive behavioral character of decision-making [23,24]. In this sense, this theory refers to the systematic use that people make of information and consideration of the consequences of their actions before carrying them out. Thus, the immediate determinant of behaviour is the intention to perform it, which is determined by three components: attitude towards the behaviour, which refers to the set of beliefs about the outcome of the behaviour and the valuation of such outcomes; subjective norms, which refer to normative beliefs about whether or not the behaviour should be performed and the motivation to comply with such pressures; finally, the perception of behavioral control, which refers to whether the person has all the power to make decisions and act accordingly in a given situation [25,26]. Thus, for an individual to develop a certain behaviour, it is necessary that he or she has a positive attitude towards the behaviour, valuing positively the state of well-being that it generates. In addition, the social context closest to the individual must positively promote this type of behaviour and even display valuable and positive attitudes, and furthermore, the person must be in control at all times of the behaviour that he or she is developing [27].

Despite the importance that the Theory of Planned Behaviour may have in helping to understand the development and establishment of young people’s behaviors, we have no evidence of studies during the Primary Education stage, although we do have evidence of studies at the Secondary Education stage. In this regard, several studies have shown how the Theory of Planned Behaviour has been positively related to behaviors related to physical activity habits [27,28,29] and balanced eating habits [30,31] and negatively related to maladaptive habits, such as cigarette smoking [32].

Thus, the present study aimed to analyze the influence of social context (parents and teachers) on basic psychological needs (frustration and satisfaction), motivation towards PE classes, attitude, subjective norms and behavioral control, eating habits intention and Mediterranean diet. The following hypotheses were proposed: (1) A social context (parents and teacher) that promotes autonomy support will positively predict psychological need satisfaction and negatively predict psychological need frustration. In contrast, a social context (parents and teacher) that promotes a controlling style will positively predict psychological need frustration and negatively predict psychological need satisfaction. (2) Satisfaction of psychological needs will positively predict motivation towards PE classes. In contrast, frustration of psychological needs will negatively predict motivation towards PE classes. (3) Motivation towards PE classes will positively predict subjective norms, attitude and behavioral control, which, in turn, will positively predict intention to maintain healthy eating habits. (4) Finally, intention will predict Mediterranean diet.

## 2. Materials and Methods

### 2.1. Participants

The number of primary school students who decided to participate in the study was 1113 (511 boys and 602 girls), aged between 9 and 12 years old (M = 10.78; SD = 0.66). The students were enrolled in different schools in the province of Burgos (Spain). Of the participants in the study: 671 were in public schools, 262 were in private schools and 180 were in state schools.

### 2.2. Measurements

Scale of Perceived Teacher Support [27]. It is a Likert-type scale (from 1 (strongly disagree) to 7 (strongly agree)) with two factors: autonomy support (12 items) and psychological control (7 items). This scale assesses, from the student’s perspective, perceived teacher support and control.

Scale of Perceived Support by Parents [27]. This is a Likert-type scale (from 1 (strongly disagree) to 7 (strongly agree)) with two factors: autonomy support (12 items) and psychological control (7 items). This scale assesses, from the student’s perspective, perceived parental support and control.

Scale of Basic Psychological Needs Satisfaction in Physical Education [33]. This is a Likert-type scale (from 1 (strongly disagree) to 7 (strongly agree)) with four factors: competence (4 items), autonomy (4 items), novelty (6 items) and relatedness (4 items). This scale assesses, from the student’s perspective, basic psychological needs satisfaction in PE.

Scale of frustration of basic psychological needs in physical education [34]. It is a Likert-type scale (from 1 (strongly disagree) to 7 (strongly agree)) with four factors: competence (4 items), autonomy (4 items), novelty (5 items) and relatedness (4 items). This scale assesses, from the student’s perspective, basic psychological needs frustration in PE.

Motivation in physical education. The Spanish version is used to analyze student motivation [35]. The scale is a Likert-type scale (from 1 (not true) to 7 (completely true)) with six factors: intrinsic motivation (4 items), integrated regulation (4 items), identified regulation (4 items), introjected regulation (4 items), external regulation (3 items) and amotivation (4 items). This scale assesses, from the student’s perspective, the motivation in physical education classes.

Social cognition and intention: The scale from the TPB, which has been used successfully in several studies [30,31], was used. The scale consists of four factors: subjective norm, intention, perceived behavioral control and attitude. The responses given by the students were on a Likert-type scale (strongly disagree (1) to strongly agree (7)), except for one item of the subjective norm factor (no control (1) to strong control (7)). This scale assesses, from the students’ perspective, their future behavioral predisposition towards balanced eating.

Balanced diet: The Spanish version of the scale linked to the Mediterranean diet [36] was used. This scale consists of 16 items, with an overall score ranging from 0 to 12.

### 2.3. Procedure

Before starting the study, contact was made with the Bioethics Committee of the University of Almeria in order to obtain its approval. Once approval was obtained (Ref. UALBIO 2020/008), the management teams of various educational centers were contacted in order to obtain their collaboration in order to carry out the study and to inform them of the objectives of the study. Subsequently, contact was established with the students of the educational centers to request their participation in the study, for which they had to provide informed consent, detailing the objectives of the study, signed by the parents or legal guardians. The questionnaires were completed individually and on paper at the beginning of the PE lessons. In addition, a member of the research group was present to answer any questions from the participants.

The completion of the questionnaires took around 25 min, and all ethical procedures established in the Helsinki Declaration were respected at all times.

### 2.4. Data Analysis

In order to achieve the objectives of the study, several statistical analyses were necessary. First, the data were analyzed descriptively, calculating mean standard deviation and bivariate correlations. Subsequently, the reliability of the Cronbach’s Alpha, Omega coefficient and AVE factors were analyzed (Table 1). Finally, structural equation modelling was carried out in order to analyze the predictive relationships between the study factors. The statistical programs SPSS v25 and AMOS v22 were used for these analyses.

**Figure 1:**
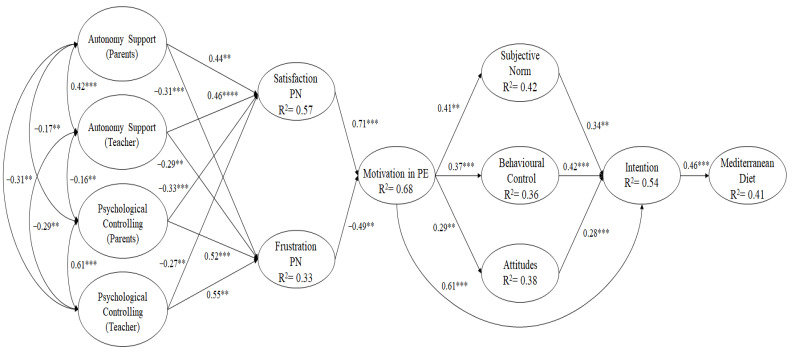
Structural Equation Model. Note: ** *p* < 0.01, *** *p* < 0.001, **** *p* < 0.0001.

The hypothesized model (Figure 1) was analyzed using the maximum likelihood method, as it takes into account the non-normal distribution of the data and is also the most suitable for Likert scales [37]. Next to the maximum likelihood method, a bootstrapping of 6000 interactions was used [38]. The following fit indices were used to define the model as good [39]: the Root Mean Square Error of Approximation (RMSEA), with its 90% confidence interval (CI), should have been found with values below 0.06, indicating an adequate and excellent fit. The incremental CFI (Comparative Fit Index), IFI (Incremental Fit Index) and TLI (Tucker–Lewis Index) with values above 0.95 were also considered adequate and excellent indices. The χ2/df index was considered adequate and excellent when it was between 2 and 3. Finally, the SRMR (Standardized Root Mean Square Residual) with values below 0.08 was also considered adequate and excellent.

## 3. Results

### 3.1. Descriptive Statistics, Reliability Analysis and Bivariate Correlations

Table 1 shows the descriptive statistics, bivariate correlations and reliability analysis. The correlations showed a positive score between those factors that showed a closer relationship, while those factors that were more distant showed a negative correlation. Reliability analysis through Cronbach’s alpha and the Omega coefficient showed a score above 0.70. In terms of discriminant validity (AVE), the score was above 0.60, reflecting the non-existence of overlap between the factors.

### 3.2. Structural Equation Model Analysis

The hypothesized models (Figure 1) tested through structural equation modelling were adequate: χ^2^ (301, *n* = 1113) = 867.78, χ^2^/df = 2.88, *p* < 0.001, TLI = 0.96, IFI = 0.96, CFI = 0.96, RMSEA = 0.063. (IC 90% = 0.057–0.069), SRMR = 0.051. The predictive relationship between each of the factors was examined through standardized regression weights, the results of which are as follows.

(1) Teacher-perceived interpersonal control style showed positive effects on psychological need frustration (β = 0.55, *p* < 0.01) and negative effects on basic psychological need satisfaction (β = −0.27, *p* < 0.01). In contrast, teacher autonomy support showed positive effects on basic psychological need satisfaction (β = −0.29, *p* < 0.01) and negative effects on psychological need frustration (β = 0.46, *p* < 0.001).

(2) Parental-perceived interpersonal control style showed positive effects on psychological need frustration (β = 0.44, *p* < 0.01) and negative effects on basic psychological need satisfaction (β = −0.31, *p* < 0.001). In contrast, parental autonomy support showed positive effects on basic psychological need satisfaction (β = 0.52, *p* < 0.01) and negative effects on psychological need frustration (β = −0.33, *p* < 0.001).

(3) Frustration of psychological needs showed negative effects on motivation towards PE classes (β = −0.49, *p* < 0.01). However, satisfaction of psychological needs showed positive effects on motivation towards PE lessons (β = 0.71, *p* < 0.001).

(4) Motivation towards PE classes showed positive effects on attitudes (β = 0.29, *p* < 0.01), behavioral control (β = 0.37, *p* < 0.001), subjective norms (β = 0.41, *p* < 0.01) and intention (β = 0.61, *p* < 0.001).

(5) Intention to follow a healthy and balanced diet was positively predicted by attitudes (β = 0.28, *p* < 0.001), behavioral control (β = 0.42, *p* < 0.001) and subjective norms (β = 0.34, *p* < 0.01).

(6) Intention to follow a healthy and balanced diet showed positive effects on the Mediterranean diet (β = 0.46, *p* < 0.01).

## 4. Discussion

The present study aimed to analyze the influence of parents and the PE teacher on the basic psychological needs, motivation and intentional eating habits of school-aged children. In this sense, PE classes are a discipline that can be fundamental in order to strengthen future adaptive behaviors related to healthy and balanced eating [40,41,42]. However, the studies in the field of Primary Education are certainly laconic, focusing mainly on the effect of satisfying the psychological needs and motivation of students on the classroom climate (e.g., fun, boredom, etc.), ignoring the instrumental objective of the subject. In this sense, it is in childhood that many of the behaviors that will take place during adolescence and adulthood are established, which is why the present study focused on this stage [43].

The results of the study showed how the social context exerts a significant influence on the satisfaction and frustration of basic psychological needs, in such a way that support for autonomy has been positively related to satisfaction and negatively related to frustration, with psychological control being the opposite. These results are in accordance with the postulates of SDT [16] and with the results achieved in several studies in the context of Secondary Education [44,45], since in Primary Education, there is no evidence of these relationships presented in the present study. Thus, the results presented here highlight the negative influence that the social context can have on the perceptual development of the environment and the psychological development of young people [46]. It is therefore necessary to create a positive classroom climate based on personal introspection and less on external demands and pressures [47].

On the other hand, basic psychological needs were positively related to motivation, whereas psychological need frustration had a negative influence on motivation. These results are similar to several studies in the context of primary education, but only taking into account the variables of psychological need satisfaction and motivation [21,22]. However, in the context of Secondary Education, there are several studies that endorse the results achieved in the present study [47,48,49]. In this sense, a study conducted by Trigueros and Navarro [50], with secondary school students aged 12–16 years, showed that satisfaction of psychological needs positively predicted motivation, while frustration exerted a negative influence. These results are in line with the postulates of SDT [16], endorsing that if students are presented with achievable challenges, have a good classroom climate and have room for self-decision, they will be more receptive and involved in classroom dynamics [17].

In addition, the results showed that motivation towards PE lessons was positively related to attitude, subjective norms, behavioral control and intention, and this, in turn, was positively related to Mediterranean diet. It is difficult to compare these results with studies in primary education. However, at the secondary school level, studies have shown that a high internal motivation towards PE classes is positively related to attitude, intention to be physically active and adopting a positive predisposition towards the Mediterranean diet as a result of a positive mental representation of the Mediterranean diet. In this sense, a study conducted by Lirola et al. [51] in a population of adolescents showed how motivation towards PE classes has a positive influence on attitude, behavioral control and subjective norms, favoring healthy and balanced eating. In the same way, but with regard to the practice of physical activity, a study carried out in a population of adolescents by Trigueros et al. [8] analyzed how motivation towards PE classes was positively related to attitude, behavioral control and subjective norms, and, in turn, attitude, behavioral control and subjective norms showed a positive relationship with regard to the intention to practice PA.

However, the results achieved in the present study are not without a number of limitations: (a) the use of self-reported questionnaires severely limited the information to be captured, although it allowed for a greater amount of information to be collected from several subjects; (b) the population participating in the study came from the same geographical location, which limits the overall representativeness of the participants; (c) the present study ignored the influence of other factors, such as emotional factors, which play a fundamental role in the adoption of behaviour. In this sense, emotions play a precursor role to motivation in judging the facts of the environment.

## 5. Conclusions

The results shown in this study confirm the clear reciprocity between SDT and TPB. In this way, the influence of the social context is key in the perceptual–psychological development of young people and in the reinforcement of behaviurs related to the healthy and balanced diet that the Mediterranean diet represents.

## Figures and Tables

**Table 1:** Descriptive statistics, internal consistency analysis and bivariate correlations.

Factors	*M*	*SD*	α	ω	AVE	1	2	3	4	5	6	7	8	9	10
1. Autonomy Support (Parents)	5.31	1.11	0.88	0.89	0.66		−0.33 **	0.41 ***	−0.21 **	0.42 ***	0.31 **	0.25 **	0.22 **	0.32 **	0.59 **
2. Psychological Control (Parents)	2.12	1.03	0.83	0.85	0.69			−0.39 **	0.55 ***	−0.33 **	−0.19 *	−0.14 **	−0.38 **	−0.24 *	−0.12 **
3. Autonomy Support (Teacher)	5.40	1.15	0.81	0.83	0.68				−0.30 *	0.49 ***	0.28 **	0.28 **	0.32 **	0.33 **	0.45 **
4. Psychological Control (Teacher)	2.07	1.04	0.84	0.85	0.71					−0.29 **	−0.39 **	−0.30 *	−0.20 **	−0.23 *	−0.26 **
5. Motivation in PE	16.61	7.32	-		-					-	0.44 ***	0.51 **	0.37 **	0.38 **	0.37 **
6. Attitude	5.11	1.88	0.80	0.82	0.72						-	0.43 **	0.40 ***	0.52 **	0.58 **
7. Behavioural Control	4.33	1.33	0.82	0.83	0.64							-	0.45 ***	0.60 ***	0.61 **
8. Subjective Norms	5.02	1.72	0.86	0.87	0.69								-	0.52 **	0.47 ***
9. Intention	5.43	1.22	0.84	0.86	0.67									-	0.61 ***
10. Mediterranean Diet	7.83	1.01	0.83	0.86	0.66										-

* *p* < 0.05; ** *p* < 0.01, *** *p* < 0.001. Note: M = Mean; SD = Standard Deviation; α = alpha de cronbach.

## Data Availability

Not applicable.
